# TCM Syndrome Recognition Model of Type 2 Diabetes Mellitus in Shanghai Based on TCM Inquiry Information

**DOI:** 10.1155/2022/2843218

**Published:** 2022-03-15

**Authors:** Chunlei Hou, Yanjie Cui, Ying Xu, Yiqin Wang, Yiming Hao

**Affiliations:** Shanghai Key Laboratory of Health Identification and Assessment, Laboratory of Traditional Chinese Medicine Four Diagnostic Information, Shanghai University of Traditional Chinese Medicine, Shanghai 201203, China

## Abstract

**Background:**

Type 2 diabetes mellitus (T2DM) has become one of the serious public health problems in China, and it affects the quality of survival of patients significantly. The long-term monitoring and early warning of T2DM and its complications should be paid attention to. Artificial diagnosis of T2DM in traditional Chinese medicine (TCM) is subjective and unrepeatable at the present stage. It is important to develop standardized collection and objective analysis methods of TCM inquiry. Therefore, we are interested in establishing syndrome recognition models.

**Objective:**

The establishment of the TCM syndrome recognition model of T2DM is helpful to the syndrome diagnosis of the disease, and the clear diagnosis of the syndrome is the prerequisite for the effective treatment of the disease by TCM. At present, there are few studies on syndrome recognition models of T2DM. Therefore, based on the inquiry information of TCM, we establish a latent structure model (LSM) of T2DM syndrome in Shanghai, hoping to provide services for the health management of TCM for diabetes in the future.

**Methods:**

A total of 587 effective samples of T2DM patients in Shanghai were collected. The gender, course of disease, and chronic complications were analyzed by one-way ANOVA and frequency analysis. Inquiry symptoms that could be included in the model were screened. In the study, 30 symptoms were used as observable variables to analyze the symptom information of TCM inquiry, and then, the TCM syndrome recognition model was established.

**Results:**

The clinical syndromes of patients with T2DM in Shanghai were mixed with deficiency and excess. The LSM divided the pattern of the disease into qi-yin deficiency pattern, yin deficiency pattern, qi deficiency pattern, and qi stagnation pattern. These patterns were mainly located in the spleen and stomach, liver, and kidney.

**Conclusion:**

The results of the syndrome classification of T2DM by LSM are basically consistent with the TCM clinical characteristics of the disease, which could reflect its main etiology and pathogenesis. The LSM of TCM inquiry syndrome diagnosis of T2DM confirmed the objectivity of TCM syndrome differentiation to some extent, and it will lay a foundation for the development of a mobile APP for TCM health management of T2DM.

## 1. Introduction

According to the International Diabetes Federation in 2019, there were 463 million diabetes patients between the ages of 20 and 79 worldwide, of which about 4.2 million died from diabetes or its complications only in 2019, accounting for 11.3% of all global deaths. At present, China has become the “largest country” of diabetes, with 116.4 million adult patients (20–79 years old), in which 90% are type 2 diabetes mellitus (T2DM) showed by another study [1]. Thus, T2DM has become one of the serious public health problems in China. T2DM in the middle and later stages of the disease will continue to develop a variety of chronic complications, threatening the health and quality of life of patients. Therefore, carrying out the research on the prevention and treatment of diabetes and realizing the early diagnosis, prevention, and treatment of type 2 diabetes are very important in order to improve the health level of Chinese people and reduce the mortality rate of patients and the national medical burden.

Syndrome differentiation and treatment is the essence of traditional Chinese medicine (TCM). In TCM syndrome differentiation, the inquiry is extremely important, and a lot of clinical information of patients can only be obtained through inquiry, especially those diseases with only conscious symptoms and lack of objective signs. However, to a certain extent, acquiring inquiry data depends on the personal experience, diagnostic skills, cognitive level, and thinking ability of doctors, so that the strong subjectivity, the poor repeatability, and the lack of unified implementation standards make it difficult to unify syndrome differentiation. In recent years, many researchers have established and explored to the standardized collection of TCM inquiry information and the exploration of objective analysis methods, which has made some progress in the objective, standardized, and procedural research of TCM inquiry. Among them, the latent structure model (LSM) proposed by Zhang [2] could analyze the information of TCM inquiry and establish a TCM syndrome recognition model. The model is the result of the multidimensional division of the population from various perspectives based on the characteristics of the data itself, which has strong objectivity and quantification. On this basis, the collection of patient information with TCM inquiry scale, wearable and portable tongue photography equipment, pulse diagnosis bracelet [3], and other objective tools of TCM four diagnosis will help to detect disease progress at low cost and provide a more efficient and fast channel for information feedback and disease follow-up between doctors and patients.

Therefore, taking diabetes as the starting point, we used the diabetes TCM inquiry scale to collect patients' clinical symptom information and established T2DM TCM inquiry database, so that we could analyze the data and construct the latent structure recognition model of TCM syndrome, which would be beneficial to the objective arrangement of the clinical data and rapid diagnosis, lay a research foundation for the objectification of TCM inquiry, and provide a theoretical basis for the realization of T2DM TCM health management mobile service.

## 2. Materials and Methods

### 2.1. Samples

All samples were collected from January 2014 to November 2020 in Yueyang Integrated Traditional Chinese and Western Medicine Hospital and Shuguang Hospital.

### 2.2. Ethical Approval

The study was approved by the Ethics Committee of Shanghai University of TCM in China in January 2014 and performed in accordance with the Declaration of Helsinki. All the subjects had signed informed consent agreements before collecting samples.

### 2.3. Criteria

#### 2.3.1. Diagnostic Criteria

Glycosylated hemoglobin (HbA1c) ≥ 6.5%, refer to the American Diabetes Association guidelines for Diabetes diagnosis and treatment (2014). The trial was conducted in accordance with the methods certified by the American HbA1c Standardization Program and standardized with the detection of diabetes control and complication studies; or fasting blood glucose ≥7.0 mmol/L, defined as no calorie intake for at least 8 hours. Or oral glucose tolerance test 2 hours blood glucose ≥11.1 mmol/L, according to the standard of World Health Organization, using 75 g anhydrous glucose dissolved in water as glucose load; or in patients with typical symptoms of hyperglycemia or hyperglycemia crisis, random blood glucose ≥11.1 mmol/L. If there is no definite hyperglycemia, the results should be confirmed by repeated tests.

#### 2.3.2. Inclusion Criteria

Patients who (1) met the diagnostic criteria of T2DM, (2) were between 30 and 80 years old, and (3) were volunteered to cooperate with the study and signed the informed consent form were included.

#### 2.3.3. Exclusion Criteria

Patients who (1) had gestational diabetes or diabetes with pregnancy, (2) psychopath, (3) with other serious primary diseases were excluded; and (4) had acute metabolic disorders such as diabetic ketoacidosis and complicated infection in recent 1 month.

### 2.4. TCM Inquiry Information Collection

The clinical cases were collected by using the TCM inquiry of the T2DM scale developed by our research group, and the scale (Copyright Bureau of Shanghai Registration Number: 09-2011-A-118) had passed the test of reliability and validity. As the objectivity of TCM clinical consultation data is largely affected by the subjective factors of both doctors and patients, in order to ensure the unity of collection standards in the process of investigation, members of the collection team were trained regularly and discussed on typical cases to ensure the standardization and consistency of the information collected as far as possible.

The symptom information of all inquiries was assigned to “1” and “0”, respectively, according to their “yes” and “no”. Data input was used by EpiData3.1 software.

### 2.5. Statistical Analysis

IBM SPSS Statistics19.0 statistical software was used for statistical analysis. The data were analyzed by one-way ANOVA and frequency analysis.

**Figure 1:**
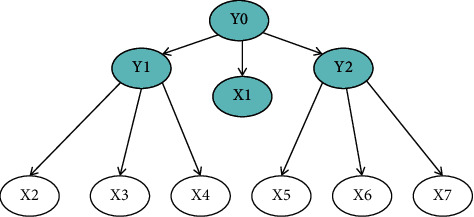
In the HLC mode, *Y*_0_, *Y*_1_, and *Y*_2_ are latent variables, and *X*_1_ and *X*_2_…*X*_7_ are observable variables.

LSM was used to construct the TCM syndrome recognition model of T2DM. Hierarchical latent class model, referred to as HLC model, is a special latent variable model [4]: (1) The network structure is a nontrivial rooted tree, in which the root node has at least two child nodes. (2) All leaf nodes represent observable variables, so they are also called observable nodes. All inner nodes represent latent variables, so they are also called latent nodes.  Figure 1 shows an HLC model.

There are many latent variables in the HLC model, and each latent variable corresponds to a clustering method. We used the double hill climbing (DHC) algorithm. That is, the model optimization problem was decomposed into potential learning problem and premodel learning problem. DHC did not search in the space of all regular HLC models directly but divided the space into two levels by using potential learning and premodel learning and searches in each level, respectively.

The analysis tool used in this paper was Lantern5.0 software. HLCM is a learning toolbox for latent structure analysis of JAVA language written by Professor Lianwen Zhang of Hong Kong University of Science and Technology. This toolbox consists of the following three parts:

  Hlcm.zip//Learning the encapsulated classes in the package
  LearnHLCM.Java//Running main program
  SampleData.Txt//Providing reference data format

The operation of the software needs to download and install the Java running environment jdk tool and set the environment variables after the download and installation. The running platform can make use of the DOS command window of the system. Before using the HLCM toolbox, we need to rewrite our format according to the format required by the tool, and during the calculation, the resulting model data are saved in a separate file.

### 2.6. Model Evaluation

Bayesian information criterion [5] (BIC) is the score matched with the optimal model in the process of running the DHC. The quality of HLC can be measured by the BIC score. On the one hand, the model is required to fit the data and reveal the laws implied in the data. On the other hand, the model is required to be as simple as possible. In the process of operation, the fitting degree between the model and data was improved continuously, so that the BIC score increased gradually. When the model becomes too complex to meet the requirements, the BIC falls instead of rises. Then, the process stops. The BIC score was measured by a negative number. In general, the higher the BIC score, the better the model.

## 3. Results

### 3.1. Summary of Demographics and Clinic Characteristics of Samples

A total of 587 T2DM patients were included in this study, in which the oldest patient was 89 years old, and the youngest patient was 24 years old. The average age was 63.35 ± 11.14 years old, including 520 patients aged 50 or above, accounting for 88.59% of all patients. The sex, course of disease, abnormality of routine physical and chemical indexes, and chronic complications are detailed in Table 1.

### 3.2. Screening Symptom Information for TCM Inquiry of T2DM

Before the recognition model of TCM syndrome of T2DM is established, it is necessary to screen the inquiry information. The principle of screening the inquiry information of T2DM is that it should reflect not only the clinical characteristics of diabetes in TCM but also the theory of syndrome differentiation of T2DM in TCM and fully embody the guiding role of TCM theory and TCM experts. The method of screening variables is combined with the frequency analysis of T2DM inquiry information and the scoring of TCM experts (3 deputy senior titles and above).

According to the frequency analysis of inquiry symptom information and the ranking of expert scores from high to low, 30 inquiry variables were determined to participate in the modeling. These symptoms included fatigue, thirst with a liking for fluids, soreness and pain of waist and knees, dizziness, difficulty to fall asleep, palpitation, easy to wake up after sleep, chest distress, irritability, dry stool, spontaneous sweating, bitter taste in the mouth, thin and sloppy stool, loss of appetite, fear of chills, tinnitus and deafness due to chronic illness or old age, frequent nocturnal urination, blurred vision, epigastric distention, itchy skin, numbness of hands and feet, anxiety, night sweats, abdominal distention, edema, fear of heat, shortness and less of breath, hiccup and belching, dry, itchy and painful on eyes, and sore mouth and tongue (Table 2).

### 3.3. Latent Structure Model of T2DM Syndrome

**Figure 2:**
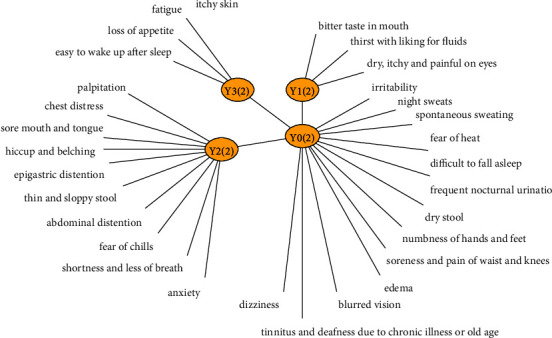
In the latent structure model of T2DM syndrome, *Y*_0_(2), *Y*_1_(2), *Y*_2_(2), and *Y*_3_(2) are latent variables, and fatigue, thirst with a liking for fluids, and soreness and pain of waist and knees, sore mouth and tongue are observable variables.

Thirty symptoms were taken as observable variables, and four recessive variables were obtained after model learning, which were recorded as *Y*_0_, *Y*_1_, *Y*_2_, and *Y*_3_, respectively, as shown in Figure 2. The BIC score of our study was −6318.41.

### 3.4. Comparison between LSM of T2DM Syndrome and TCM Theory in Qualitative and Quantitative Aspect

#### 3.4.1. Interpretation of TCM Theory to Model

There were four latent variables in the model. Each latent variable represented the partition of a data sample, and each partition was composed of two or more latent classes. The qualitative evaluation of the model was to explain the meanings of these partitions and latent classes from the perspective of TCM. Each latent variable had two inseparable aspects: on the one hand, it represented a partition; on the other hand, it represented the mechanism of partition. The cases collected and analyzed in our subject were all patients with T2DM, which were consistent with the dysfunction of qi, blood, yin, and yang in TCM.

With setting the cumulative information coverage to 95% in the software, this study derived a lot of symptom information (observable variables) covered by latent variables and exported-related information curves plot.

**Figure 3:**
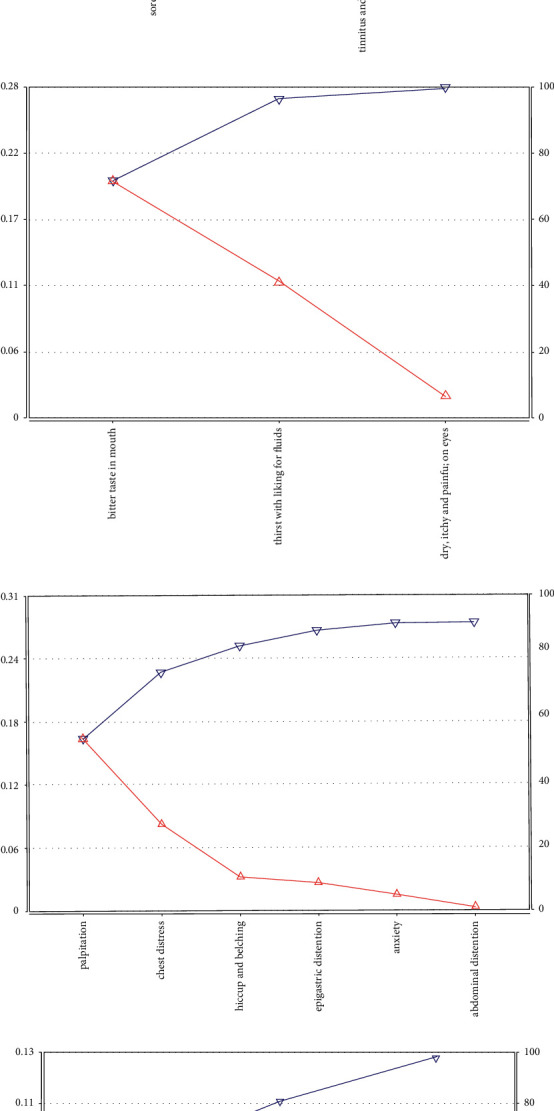
Observable variable mutual information and information coverage curve. (a) Observable variable mutual information and information coverage curve of latent variable *Y*_0_. (b) Observable variable mutual information and information coverage curve of latent variable *Y*_1_. (c) Observable variable mutual information and information coverage curve of latent variable *Y*_2_. (d) Observable variable mutual information and information coverage curve of latent variable *Y*_3_.

In the information graph (Figure 3(a)), it could be seen that the latent variable *Y*_0_ affected the appearance of variables such as irritability, dizziness, soreness, and pain of waist and knees, spontaneous sweating, blurred vision, difficulty to fall asleep, numbness of hands and feet, tinnitus, and deafness due to chronic illness or old age, fear of heat, and night sweats. Yin deficiency and internal heat caused being afraid of heat. Internal heat disturbed the heart mind, which led patients to be irritable. The deficiency of liver and kidney yin kept eyes out of being moisturized and then blurred vision appeared. Waist tendons and veins lost their nourishment because of loss of kidney essence, so the waist and knees were pained. Abnormal renal function may lead to the lesion of ear function. Chronic illness or old age made kidney yin deficiency, so tinnitus and deafness came out. Liver yin deficiency or qi deficiency, which resulted in blood deficiency or qi deficiency, failed to transport and transform the refined substance, and finally, numbness of hands and feet appeared. Kidney yin deficiency produced internal heat and disturbed heart fire. Heart fire was hyperactive, so it was difficult to fall asleep. Yin deficiency and internal heat forced fluid to leak out, which caused night sweats. Qi deficiency made head and eyes malnutrition, and dizziness appeared. Defensive Qi instability caused spontaneous sweating. Therefore, *Y*_0_ could be interpreted as a “qi-yin deficiency pattern” by the theory of TCM.

The latent variable *Y*_1_ affected the occurrence of bitter taste in the mouth, thirst with a liking for fluids, and dry, itchy, and painful on eyes. Yin deficiency of liver and kidney caused deficiency of fire. Liver qi was nonflowing, so bile spilling out caused a bitter taste in the mouth. Yin deficiency and internal heat consumed body fluid, which caused thirst with a liking for fluids. Body fluid could not moisten the eyes, so the eyes were dry and painful. Therefore, *Y*_1_ could be interpreted as “yin deficiency” by the theory of TCM (Figure 3(b)).

The latent variable *Y*_2_ affected palpitation, chest distress, hiccup and belching, epigastric distension, irritability, and abdominal distention. Heart qi stagnated in the heart and chest, or phlegm and blood stasis blocked, which caused heart pulse obstruction, so palpitation and chest distress appeared. Spleen and stomach qi stagnation brought about abnormal transport of cereal nutrients, so epigastric distension and abdominal distension appeared. The stomach was appropriate to fall, so when the stomach qi reversed upward, hiccup and belching came out. When liver qi was depressed, qi was stagnated, which led to irritability. Therefore, *Y*_2_ could be interpreted as “qi stagnation” by the theory of TCM (Figure 3(c)).

The latent variable *Y*_3_ affected the appearance of loss of appetite, being fatigued, and difficulty to fall asleep. Spleen and stomach qi deficiency caused abnormal transportation function, so the loss of appetite appeared. Qi deficiency could not help qi and blood distributing throughout the body, which led to viscera function decline, so the patients were fatigued. Deficiency of heart and spleen qi made qi and blood insufficient transformation. Heart blood deficiency caused heart loss of nourishment, so the patients were easy to wake up after sleep. Therefore, *Y*_3_ could be interpreted as “qi deficiency” by the theory of traditional Chinese medicine (Figure 3(d)).

The corresponding relationship between latent variables in the model and TCM syndromes is shown in Table 3.

#### 3.4.2. Evaluation of Quantitative Content of Model

The quantitative content of the model has two aspects: (1) The number of latent variables in the model is 4. (2) The class conditional probability distribution is used to quantitatively describe the relationship between variables.

The partition represented by latent variables in the latent structure model is a probabilistic soft partition. A thing may belong to a certain class with a certain probability, and it can also belong to another class with a certain probability. In order to grasp the characteristics of each class in probability soft division, it is necessary to investigate their class conditional probability distribution. We gave the quantitative relationship between the latent variables and the observable variables by the class conditional probability distribution *P*(*X* = 1│*Y* = *S*_1_) and *P*(*X* = 1│*Y* = *S*_0_). The conditional probability distribution of the two latent classes *Y*_0_ = *S*_1_ and *Y*_0_ = *S*_0_ of *Y*^0^ is shown in Table 4. There were two values of *S*_1_ and *S*_0_ in the group of deficiency of both qi and yin in *Y*_0_, indicating that model M had divided the population of deficiency of both qi and yin into two categories, namely “appearing” (*S*_1_) or “not appearing” (*S*_0_). The column *P*(*X* = 1│*Y*_0_ = *S*_1_) gave the probability that the observable variable valued for 1(that is, the occurrence of symptoms) in the latent *Y*_0_ = *S*_1_, while the column *P*(*X* = 1│*Y*_0_ = *S*_0_) gave the probability that the observable variable valued for 1 in the latent *Y*_0_ = *S*_0_. According to Table 4, the main difference between the two latent categories was that the probability of occurrence of all 11 symptoms in *Y*_0_ = *S*_1_ was higher, while in *Y*_0_ = *S*_0_, the probability was much lower. Especially, fear of heat was close to zero. The relationship between “a deficiency of both qi and yin” and “irritability, dizziness, soreness, and pain of waist and knees” was quantitatively described by conditional probability, which was consistent with the theory of traditional Chinese medicine.

As could be seen from Table 5, in the category of *Y*_1_ = *S*_1_, there was a higher probability of bitter taste in the mouth, thirst with a liking for fluids, and dry, itchy, and painful on eyes, while in the category of *Y*_1_ = *S*_0_, there was a certain probability of thirst with a liking for fluids. The probability of bitter taste in the mouth and dry, itchy, and painful on eyes was lower. It showed that the symptoms of bitter taste in the mouth and dry, itchy, and painful on eyes also had a certain probability in other patterns. It was quite consistent with the theory of TCM because the relationship between TCM patterns and symptoms was not a one-to-one correspondence. The combination of symptoms played an important role in pattern differentiation.

Although TCM theory did not strongly confine the presence of a symptom, it implied that the symptoms depended on the change of the degree of the syndrome. For a symptom, the higher the severity of the pattern, the more likely it was to appear, and the more severe it would appear. Similarly, the class probability distributions of other latent variables in the model are shown in Tables 6 and 7

**Figure 4:**
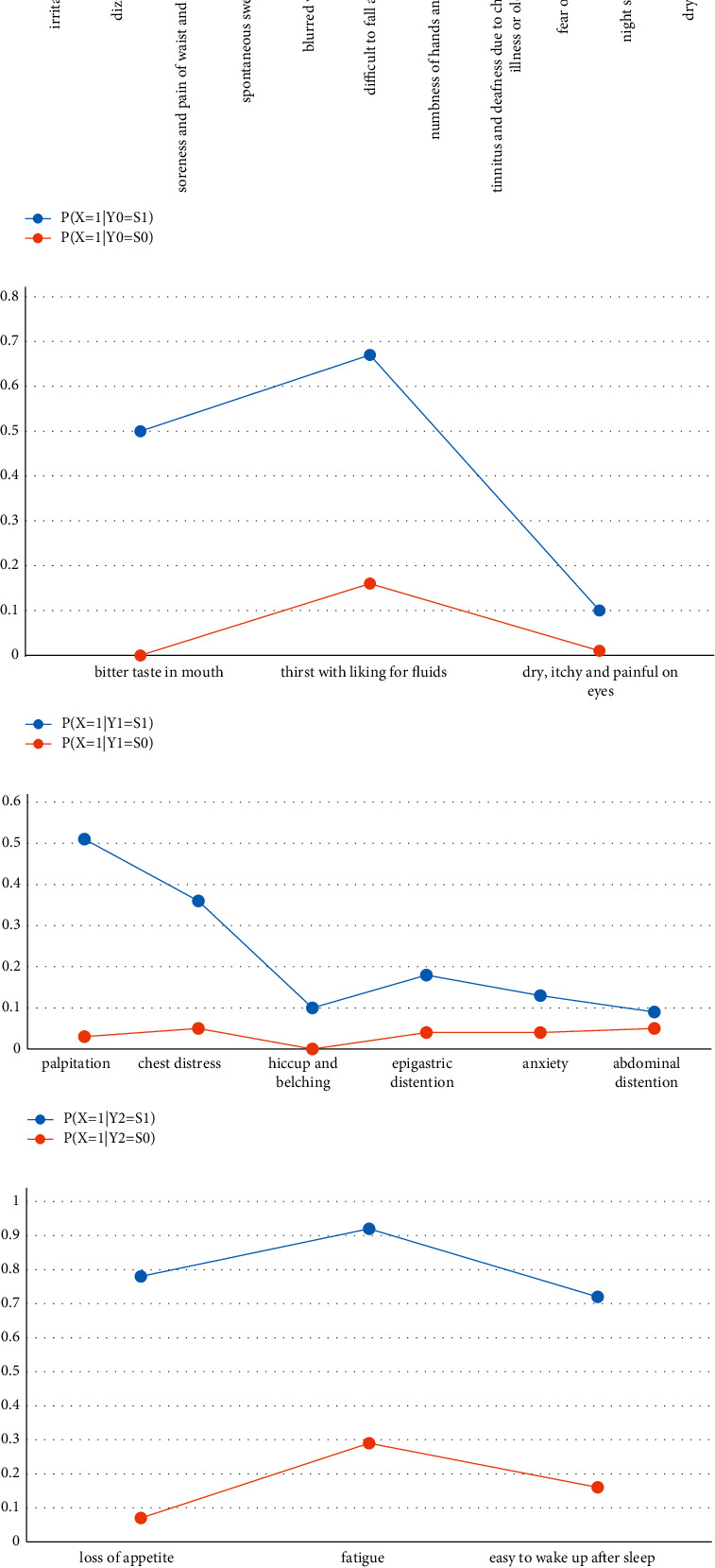
Line charts of conditional probability distributions. (a) Line charts of conditional probability distributions of two latent classes *Y*_0_ = *S*_1_ and *Y*_0_ = *S*_0_ of latent variable *Y*_0_. (b) Line charts of conditional probability distributions of two latent classes *Y*_1_ = *S*_1_ and *Y*_1_ = *S*_0_ of latent variable *Y*_1_. (c) Line charts of conditional probability distributions of two latent classes *Y*_2_ = *S*_1_ and *Y*_2_ = *S*_0_ of latent variable *Y*_2_. (d) Line charts of conditional probability distributions of two latent classes *Y*_3_ = *S*_1_ and *Y*_3_ = *S*_0_ of latent variable *Y*_3_.

In order to make the characteristics of latent classes clear, the probability distributions of these classes could be shown in a line chart (Figure 4).

## 4. Discussion

Based on the Bayesian network and probability graph model, the latent structure model reveals the latent characteristics behind observable variables by analyzing the latent class probability, conditional probability, mutual information, and information coverage and excavates the logical relationship between the observable variables and the latent variables. It is a special method proposed for the research of TCM. The basic idea is to use computers instead of human brains to analyze data and construct latent structures. Whether the symptoms of TCM appear or not is not independent of each other but related to each other. Through the observation of the occurrence of symptoms in many patients, we can find a variety of statistical relationships between them, which provide the basis for finding the latent structure behind the symptoms.

In term of TCM data exploration, we often pay special attention to the relationship and pattern among symptoms and between symptoms and patterns, which means we would pay special attention to the two-variable relationship study between latent variables and latent variables or observable variables. The theory of syndrome differentiation of TCM includes observable variables, latent variables, and latent classes. The relationship between them constitutes a latent structure. The latent structure method [6] is based on the results collected from the clinical epidemiological investigation, by which it replaced the human brain for data analysis on a computer, and constructed a model which could define the pattern well. Finally, the LSM is used to guide syndrome differentiation, which is objective to a certain extent.

Diabetes is a disease related to wasting thirst in TCM. In this study, the latent structure method was used to analyze the TCM inquiry symptom information of 587 patients with T2DM in Shanghai, and the syndrome recognition models of qi-yin deficiency pattern, yin deficiency pattern, qi deficiency pattern, and qi stagnation pattern were established, and these syndromes were mainly located in spleen and stomach, liver, and kidney.

In the modern research of TCM syndrome of diabetes, many researchers believe that the pattern of deficiency of both qi and yin is the most common pattern of T2DM [7, 8]. After a certain period of disease development, phlegm turbidity and blood stasis are still the most common signs during disease evolution [9, 10].

Xu. et al. [11] have investigated the distribution of TCM syndrome types of diabetes, and their results showed that deficiency of both qi and yin accounted for the most significant pattern, in which many of them had blood stasis during the disease. Ai et al. [12] and others found that the distribution of TCM patterns of diabetes mainly included deficiency of both qi and yin, dryness-heat, and phlegm-dampness. The research of Zhao [13] showed that the TCM patterns of diabetic patients were mainly yin deficiency and qi deficiency, accounting for 26.0% and 21.9%, respectively, which were consistent with the syndrome characteristics of qi and yin deficiency of thirst disease. Ren et al. [14] and others studied the patterns of prediabetes by the method of cluster analysis. Their results implied that the characteristics of deficiency in origin and excess in superficiality in prediabetes were obvious, and the symptoms of qi deficiency patterns such as spontaneous sweating and mental fatigue were prominent. It suggested that the qi deficiency pattern played a leading role in patients with prediabetes.

Yang et al. [15] found that patients with diabetes complicated with depression had more obvious pathogenesis characteristics of liver depression and internal obstruction of blood stasis than those without depression. Cao [16], by analyzing single syndrome statistics, found that the patients with qi stagnation patterns accounted for 27.98% of 218 diabetic patients with stable angina pectoris. Zhang [17] found that the frequency of heat in perianal abscess with T2DM was the highest, followed by yin deficiency, phlegm-dampness, and qi stagnation. According to the modern research on the epidemiological investigation of diabetes, qi-yin deficiency pattern, yin deficiency pattern, qi deficiency pattern, and qi stagnation pattern were all common clinical patterns of the disease, which showed that the latent structure model established in this study was basically in line with the clinical actual situation.

In this study, the latent variable qi and yin deficiency pattern led to dizziness. In addition to qi deficiency, the main pathogenesis of dizziness might be related to internal obstruction of phlegm and dampness disturbing clear yang or internal stagnation of blood stasis blocking the flow of qi and blood, and then, the head could not be nourished. The latent variable qi stagnation led to the emergence of palpitation, and its main pathogenesis was not only related to qi stagnation but also related to phlegm-dampness and blood stasis blocking clear yang, resulting in malnutrition of heart spirit. Latent variable qi stagnation also led to epigastric distention and abdominal distension, which might also be related to phlegm-dampness blocking middle-jiao and water accumulating. It could be seen that, in addition to the syndrome obtained by this model, phlegm-dampness syndrome and blood stasis syndrome would also appear in the development of T2DM.

The clinical manifestations related to phlegm-dampness syndrome and blood stasis syndrome are often reflected in the tongue and pulse of patients. The tongue body of patients with phlegm-dampness syndrome is fat, with greasy moss or slippery moss, and the pulse is moist or slippery. The tongue manifestation of blood stasis syndrome is purple and dark, with petechiae, ecchymosis, or sublingual collateral varicose veins. The pulse is usually a rough pulse or irregularly intermittent pulse. However, this study only used the TCM inquiry scale, which did not include the tongue, pulse, and other clinicopathologic data related to inspection and amputation.

In this study, the LSM of TCM inquiry syndrome diagnosis of T2DM confirmed the objectivity of TCM syndrome differentiation to some extent. In the future, based on the ideas and results of this study, we hope to use the “Internet Plus” technology, combined with TCM inquiry scale, tongue photography software, pulse diagnosis bracelet, and other TCM four diagnosis objective mobile devices, to lay the foundation for the research of objective disease recognition model of four diagnosis information fusion. With the development of sensor, chip, mobile Internet, and other technologies, wearable and portable devices have shown great potential. This study will contribute to the development of a mobile APP for TCM health management of T2DM, to achieve mobile monitoring, management, and early warning of the disease development for diabetes. Through the establishment of the web-based information collection system and circulation system for diabetes patients, it can help doctors obtain objective information of diabetes patients in real time, establish a new follow-up system, and provide doctors with a more convenient, fast, and efficient way of follow-up. At the same time, it reduces the difficulty for patients to obtain feedback information, increases the viscosity and compliance of follow-up, reduces the long-term complications of diabetes, and improves physical condition, quality of life, and span of patients. In addition, with the help of the “noncontact” four-diagnosis mode of mobile APP based on TCM health management, it is conducive to the collection of clinical data and rapid diagnosis of isolated patients with infectious diseases such as COVID-19, so as to reduce the medical burden of the country.

In addition, the cases included in this study were all people in Shanghai, and the distribution of TCM patterns of T2DM also had a certain regional nature. Therefore, in future research, we are going to expand the sample size and collect TCM clinical information of diabetic patients from other representative areas, so that it may constantly improve the syndrome recognition model to make it more universal.

## Figures and Tables

**Table 1:** Summary of gender, course of disease, and chronic complications of patients (*n* = 587).

Demographics and clinical information	Count	Frequency (%)
Sample number	587	N/A
Number of males in the sample	257	43.78
Number of females in the sample	330	56.22
Ratio of males to females	1 : 1.28	N/A
Average age (year)	63.35 ± 11.14	N/A
Number (percentage) of samples diagnosed this time	32	9.20
Number (percentage) of samples diagnosed for 1–3 years	101	29.02
Number (percentage) of samples diagnosed for 4–10 years	132	37.93
Number (percentage) of samples diagnosed for 10–20 years	69	19.83
Number (percentage) of samples diagnosed more than 20 years	14	4.02
Number (percentage) of samples with coronary heart disease	59	10.05
Number (percentage) of samples with kidney disease	34	5.79
Number (percentage) of samples with retinopathy	18	3.07
Number (percentage) of samples with neuropathy	5	0.85
Number (percentage) of samples with foot lesions	2	0.34
Number (percentage) of samples with complications more than two	15	2.46

**Table 2:** Inquiring symptoms of T2DM included in latent structure model after the frequency analysis of inquiry symptom information and the ranking of the expert.

Syndromes	Count (%)
Fatigue	190 (32.37%)
Soreness and pain of waist and knees	160 (27.26%)
Difficult to fall asleep	132 (22.49%)
Easy to wake up after sleep	109 (18.57%)
Irritability	91 (15.50%)
Spontaneous sweating	84 (14.31%)
Thin and sloppy stool	71 (12.10%)
Fear of chills	62 (10.56%)
Frequent nocturnal urination	59 (10.05%)
Epigastric distention	56 (9.54%)
Numbness of hands and feet	45 (7.67%)
Night sweats	42 (7.16%)
Edema	35 (5.96%)
Shortness and less of breath	27 (4.60%)
Dry, itchy, and painful on eyes	21 (3.58%)
Thirst with liking for fluids	172 (29.30%)
Dizziness	136 (23.17%)
Palpitation	130 (22.15%)
Chest distress	101 (17.21%)
Dry stool	89 (15.16%)
Bitter taste in mouth	76 (12.95%)
Loss of appetite	64 (10.90%)
Tinnitus and deafness due to chronic illness or old age	60 (10.22%)
Blurred vision	57 (9.71%)
Itchy skin	50 (8.52%)
Anxiety	43 (7.33%)
Abdominal distention	37 (6.30%)
Fear of heat	28 (4.77%)
Hiccup and belching	24 (4.09%)
Sore mouth and tongue	20 (3.41%)

**Table 3:** The corresponding relationship between latent variables in the model and TCM patterns.

*Y*_0_	Qi-yin deficiency pattern
*Y*_1_	Yin deficiency pattern
*Y*_2_	Qi stagnation pattern
*Y*_3_	Qi deficiency pattern

**Table 4:** Conditional probability distribution table of two latent class *Y*_0_ = *S*_1_ and *Y*_0_ = *S*_0_ of latent variable *Y*_0_ class.

Observable variables	*P*(*X* = 1│*Y*_0_ = *S*_1_)	*P*(*X* = 1│*Y*_0_ = *S*_0_)
Irritability	0.36	0.05
Dizziness	0.43	0.13
Soreness and pain of waist and knees	0.47	0.17
Spontaneous sweating	0.29	0.07
Blurred vision	0.22	0.04
Difficult to fall asleep	0.38	0.15
Numbness of hands and feet	0.17	0.03
Tinnitus and deafness due to chronic illness or old age	0.20	0.05
Fear of heat	0.12	0.01
Night sweats	0.13	0.04
Dry stool	0.23	0.11

**Table 5:** Conditional probability distribution table of two latent class *Y*_1_ = *S*_1_ and *Y*_1_ = *S*_0_ of latent variable *Y*_1_ class.

Observable variables	*P*(*X* = 1│*Y*_1_ = *S*_1_)	*P*(*X* = 1│*Y*_1_ = *S*_0_)
Bitter taste in mouth	0.50	0.00
Thirst with liking for fluids	0.67	0.16
Dry, itchy, and painful on eyes	0.10	0.01

**Table 6:** Conditional probability distribution table of two latent class *Y*_2_ = *S*_1_ and *Y*_2_ = *S*_0_ of latent variable *Y*_2_ class.

Observable variables	*P*(*X* = 1│*Y*_2_ = *S*_1_)	*P*(*X* = 1│*Y*_2_ = *S*_0_)
Palpitation	0.51	0.03
Chest distress	0.36	0.05
Hiccup and belching	0.10	0.00
Epigastric distention	0.18	0.04
Anxiety	0.13	0.04
Abdominal distention	0.09	0.05

**Table 7:** Conditional probability distribution table of two latent class *Y*_3_ = *S*_1_ and *Y*_3_ = *S*_0_ of latent variable *Y*_3_ class.

Observable variables	*P*(*X* = 1│*Y*_3_ = *S*_1_)	*P*(*X* = 1│*Y*_3_ = *S*_0_)

Loss of appetite	0.78	0.07
Fatigue	0.92	0.29
Easy to wake up after sleep	0.72	0.16

## Data Availability

The Ethics Committee of Shanghai University of TCM limited the measurement data used to support the results of this study in order to protect the privacy of patients. For researchers who meet the criteria for obtaining confidential data, the data of this study can be obtained from Yiming Hao (e-mail: hymjj888@163.com).
